# Reduced Immunogenicity of Induced Pluripotent Stem Cells Derived from Sertoli Cells

**DOI:** 10.1371/journal.pone.0106110

**Published:** 2014-08-28

**Authors:** Xiaoying Wang, Jie Qin, Robert Chunhua Zhao, Martin Zenke

**Affiliations:** 1 Institute for Biomedical Engineering, Department of Cell Biology, RWTH Aachen University Medical School, Aachen, Germany; 2 Helmholtz Institute for Biomedical Engineering, RWTH Aachen University, Aachen, Germany; 3 Institute of Basic Medical Sciences, Chinese Academy of Medical Sciences, School of Basic Medicine, Peking Union Medical College, Beijing, China; 4 Center of Excellence in Tissue Engineering, Peking Union Medical College Hospital, Beijing, China; National University of Singapore, Singapore

## Abstract

Sertoli cells constitute the structural framework in testis and provide an immune-privileged environment for germ cells. Induced pluripotent stem cells (iPS cells) resemble embryonic stem cells (ES cells) and are generated from somatic cells by expression of specific reprogramming transcription factors. Here, we used C57BL/6 (B6) Sertoli cells to generate iPS cells (Ser-iPS cells) and compared the immunogenicity of Ser-iPS cells with iPS cells derived from mouse embryonic fibroblast (MEF-iPS cells). Ser-iPS cells were injected into syngeneic mice to test for their *in* *vivo* immunogenicity in teratoma assay. Teratoma assay allows assessing *in* *vivo* immunogenicity of iPS cells and of their differentiated progeny simultaneously. We observed that early-passage Ser-iPS cells formed more teratomas with less immune cell infiltration and tissue damage and necrosis than MEF-iPS cells. Differentiating Ser-iPS cells in embryoid bodies (EBs) showed reduced T cell activation potential compared to MEF-iPS cells, which was similar to syngeneic ES cells. However, Ser-iPS cells lost their reduced immunogenicity *in* *vivo* after extended passaging *in* *vitro* and late-passage Ser-iPS cells exhibited an immunogenicity similar to MEF-iPS cells. These findings indicate that early-passage Ser-iPS cells retain some somatic memory of Sertoli cells that impacts on immunogenicity of iPS cells and iPS cell-derived cells *in* *vivo* and *in* *vitro*. Our data suggest that immune-privileged Sertoli cells might represent a preferred source for iPS cell generation, if it comes to the use of iPS cell-derived cells for transplantation.

## Introduction

Induced pluripotent stem cells (iPS cells) resemble embryonic stem cells (ES cells) and can differentiate into all cell types of our body [1]. iPS cells are generated from somatic cells by expression of a defined set of transcription factors, including Oct4, Sox2, Klf4 and c-Myc (OSKM) [2]. iPS cells have been successfully generated from a broad range of somatic cell types, such as fibroblasts, B lymphocytes, neural stem cells and hepatocytes [2]–[5]. iPS cells hold great potential in disease modeling, drug screening and cell-based therapy [1], [2].

iPS cells are believed to be particularly appealing as a cell source for personalized regenerative therapies, since autologous iPS cell-derived cells are expected to bypass immune rejection [6]. However, this assumption has been challenged by recent studies [7], [8]. Syngeneic iPS cells derived from mouse fibroblasts were reported to be immunogenic and rejected upon transplantation as measured by teratoma formation and lymphocytic infiltration [7], [8]. In contrast, Guha et al. [9] did not find evidence for rejection of syngeneic iPS cells and their differentiated cells after transplantation and for secondary immune responses. Further, in other studies it was found that syngeneic iPS cell-derived cells elicited only minimal immune responses or induced tolerogenic responses following transplantation [8], [10]. Yet cardiomyocytes derived from iPS cells *in* *vitro* caused significant levels of T cell infiltration after syngeneic transplantation [10]. Thus, the immunogenicity of iPS cells and iPS cell-derived cells has remained highly controversial.

An interesting question is what might cause immunogenicity of iPS cells and their differentiated progeny? The somatic cell type used for reprogramming might impact on the immunogenicity of iPS cells [11]. Human umbilical cord mesenchymal cells were used for iPS cell generation, since mesenchymal cells exhibit immune-modulatory properties [12]. Neural progenitors derived from these iPS cells showed lower immunogenicity compared to those from fibroblast-derived iPS cells. However, in this study the immunogenicity of iPS cells and iPS cell-derived cells was only investigated *in* *vitro* and several questions remained: (i) Is low immunogenicity of these iPS cells also observed *in* *vivo*? (ii) Does passage number affect iPS cell immunogenicity? (iii) Do other immune-privileged cells impact on iPS cell immunogenicity?

Testis is considered an immunologically privileged organ [13]. Testicular Sertoli cells and germ cells constitute the structural framework of the seminiferous tubules. Sertoli cells represent key players in the immune-privileged testicular environment [14]. Sertoli cell immune function was also observed in co-transplantation studies with skin or islet grafts, where grafts showed a significantly prolonged survival [15], [16]. Several mechanisms might contribute to Sertoli cell immune function. First, Sertoli cells produce immune-modulatory factors, which might contribute to the immune-privileged testicular milieu [17], [18]. Second, the physical barrier formed by adjacent Sertoli cell membranes, referred to as blood-testis barrier, is responsible for controlling and regulating the environment of developing germ cells [19], [20]. Thus, all these properties make Sertoli cells an interesting target for clinical application.

Here, we used Sertoli cells to generate iPS cells (referred to as Ser-iPS) and compared their immunogenicity with iPS cells derived from mouse embryonic fibroblasts (MEF-iPS). We systematically analyzed the immunogenicity of Ser-iPS cells and found that Ser-iPS cells indeed possess reduced immunogenicity both *in* *vivo* and *in* *vitro*.

## Materials and Methods

### Generation of Ser-iPS cells

Sertoli cells from C57BL/6 (B6) mice (age 7–10 days) were obtained as described [21] with the following modifications. Briefly, de-capsulated testis was treated with 1 mg/ml collagenase IV (Invitrogen, Carlsbad, CA) followed by 0.05% trypsin (Invitrogen) for 5 min to remove interstitial cells and germ cells, respectively. Cells were then filtered through 40 µm cell strainer and incubated in Sertoli cell medium: DMEM/F12 (1∶1) supplemented with 10% fetal calf serum (FCS) (v/v), 2 mM L-glutamine, 100 units/ml penicillin and 100 µg/ml streptomycin (all Invitrogen).

iPS cells from B6 Sertoli cells and B6 MEF were generated with retroviral vectors expressing Oct4, Sox2, Klf4 and c-Myc (OSK and OSKM) as described previously [2], [4], [22]. About 10 days after infection iPS colonies were picked and plated on MEF feeder cells with ES cell medium, in the following referred to as Ser-iPS cells. MEF-iPS cells were generated from MEF accordingly and used as a control. 86 Ser-iPS cell clones and 65 MEF-iPS cell clones were picked and eventually 19 Ser-iPS cell clones and 17 MEF-iPS cell clones were expanded. We then selected three representative Ser-iPS cell clones for further analysis: one representative 4F Ser-iPS cell clone (OSKM, clone 1) and two representative 3F Ser-iPS cell clones (OSK, clones 2 and 3). Two MEF-iPS cell clones were also selected: one representative 4F (OSKM) and one representative 3F (OSK) MEF-iPS cell clone, MEF-iPS cell clone 1 and 2, respectively. ES cell medium: DMEM (high glucose) supplemented with 15% FCS (Lonza, Basel, Switzerland), 0.1 mM β-mercaptoethanol, 100 units/ml penicillin and 100 µg/ml streptomycin, 2 mM L-glutamine, 0.1 mM nonessential amino acids and 25 mM HEPES (all Invitrogen) and 1000 U recombinant leukemia inhibitory factor (LIF; Peprotech, London, UK).

### Embryoid body (EB) assay

For spontaneous differentiation *in* *vitro*, undifferentiated iPS cells and ES cells (JM8) were trypsinized into single cells and plated on gelatin-coated dish for 40 min to remove MEF feeder cells. Cells were transferred into differentiation medium (ES cell medium without LIF) and EBs were generated in hanging drops (500 cells per 20 µl drops) in an inverted bacterial Petri dish. EBs were collected 3 days later and kept in suspension culture for another 3 days. On day 6 of differentiation, EBs were plated on 0.1% gelatin-coated dishes for further differentiation (6 days) and used in T cell co-cultures.

### RT-PCR and qRT-PCR

RNA was isolated using NucleoSpin RNA Kit (Macherey-Nagel, Dueren, Germany) and concentration was determined by NanoDrop 1000 (Thermo Scientific, Wilmington, DE). 1 µg RNA was used for reverse-transcription using High Capacity cDNA reverse transcription Kit (Applied Biosystems, Carlsbad, CA). RT-PCR was performed in thermal cycling machine (Eppendorf, Hamburg, Germany). For qRT-PCR 50 ng cDNA, fast SYBR Green PCR master mix and primers were used (Table S1). PCR reactions were performed with StepOne Real-Time PCR system (Applied Biosystems, Carlsbad, CA). Data are represented in heatmap format (MultiExperiment Viewer MeV_4_8, http://www.tm4.org) with fold change in gene expression normalized to β-actin.

### Alkaline phosphatase (AP) staining and immunofluorescence staining

AP staining was done with Alkaline Phosphatase Staining Kit II (Stemgent, Cambridge, MA) according to the manufacture’s instruction. For immunofluorescence staining, cells were grown on gelatin-coated cover-slips, fixed with 4% paraformaldehyde (PFA) (20 min, RT) and permeabilized with PBS containing 0.1% Triton X-100 (30 min, RT). Samples were stained with first antibody (14–16 hours, 4°C), washed twice with PBS and incubated with secondary antibody (1 hour, RT). Cell nuclei were stained with DAPI (0.1 µg/mL, 30 min). Samples were mounted with mounting solution (Dako, Glostrup, Denmark) and images were acquired with Axiovert 200 microscope (Carl Zeiss, Jena, Germany). The following primary antibodies were used: Oct4 and SSEA1 (1∶200; Santa Cruz Biotechnology, Santa Cruz, CA). The secondary antibody was Alexa594 Goat anti-mouse Ig (H+L) (1∶300; Life Technologies, Carlsbad, CA).

### Teratoma assay and histological analysis

1×10^6^ B6 Ser-iPS cells, MEF-iPS cells and ES cells (JM8) were injected subcutaneously into B6 mice. Four weeks later teratomas were excised, fixed in 4% PFA, embedded in paraffin and stained with rabbit polyclonal anti-CD3 antibody (1∶200 dilution, Abcam, Cambridge, UK). The secondary antibody was VECTASTAIN® ABC Reagent (Vector Laboratories, Peterborough, UK). Hematoxylin and eosin (HE) staining was performed at IZKF Immunohistochemistry Core Facility (RWTH Aachen University Hospital, Aachen, Germany). Frequency of teratoma formation was assessed as number of injections relative to teratomas formed. All experimental procedures involving mouse work were approved by the local authorities in compliance with the German animal protection law (Landesamt für Natur, Umwelt und Verbraucherschutz, LANUV NRW, Recklinghausen, Germany; reference number 8.87-50.10.35.08.138). All efforts were made to minimize animal suffering.

### Co-culture experiments and flow cytometry

CD4 T cells were obtained from spleen of B6 mice by MACS selection (Miltenyi Biotec, Bergisch Gladbach, Germany). For T cell proliferation assay, CD4 T cells were labeled with carboxyfluorescein succinimidyl ester (CFSE) and co-cultured with undifferentiated iPS cells or with EB iPS cells (day 12–17 of differentiation) at 2∶1 ratio. T cells treated with phorbol myristate acetate (PMA, 25 ng/ml) and ionomycin (0.02 mM) were used as positive control. After 5 days of co-culture, proliferation of CD4 T cells was analyzed by assessing the dilution of CFSE signal using flow cytometry (Canto II, BD Bioscience). The CD4 antibody used was PE anti-mouse CD4 (GK1.5; eBioscience, San Diego, CA).

For regulatory T cell (Treg) assay, CD4 splenic T cells were activated with PMA and ionomycin (14–16 hours) and co-cultured with undifferentiated iPS cells or with EB iPS cells (day 12–17 of differentiation) at 2∶1 ratio. After 5 days of co-culture, T cells were harvested and stained for surface markers CD4 and CD25 (FITC anti-mouse CD4, RM4-5; APC anti-mouse CD25, PC61.5; eBioscience; 30 min, 4°C). Subsequently, cells were fixed and permeabilized using the Foxp3 fixation/permeabilization buffer and intracellular Foxp3 (PE anti-mouse/rat Foxp3, FJK-16s; eBioscience) staining was carried out according to manufacturer’s instruction. The percentage of Tregs was determined by flow cytometry based on the expression of CD4, CD25 and Foxp3.

### Statistics analysis

Results are given as the mean ± standard derivation. Statistical analysis was performed using unpaired Student’s t-test. P values below 0.05 were considered to be statistically significant.

## Results

### Generation of Ser-iPS cells

Sertoli cells were obtained from testis of day 7–10 pups and showed the typical Sertoli cell morphology in culture [23] (Figure S1A). Cells expressed the Sertoli cell marker Gata4 [17] and were negative for Dazl1 [24] and Vasa [25] (germ cells), Oct4 and Nanog (spermatogonial stem cells, [26]) and Hsd3β6 (Leydig cells, [27]) (Figure S1B).

**Figure 1:**
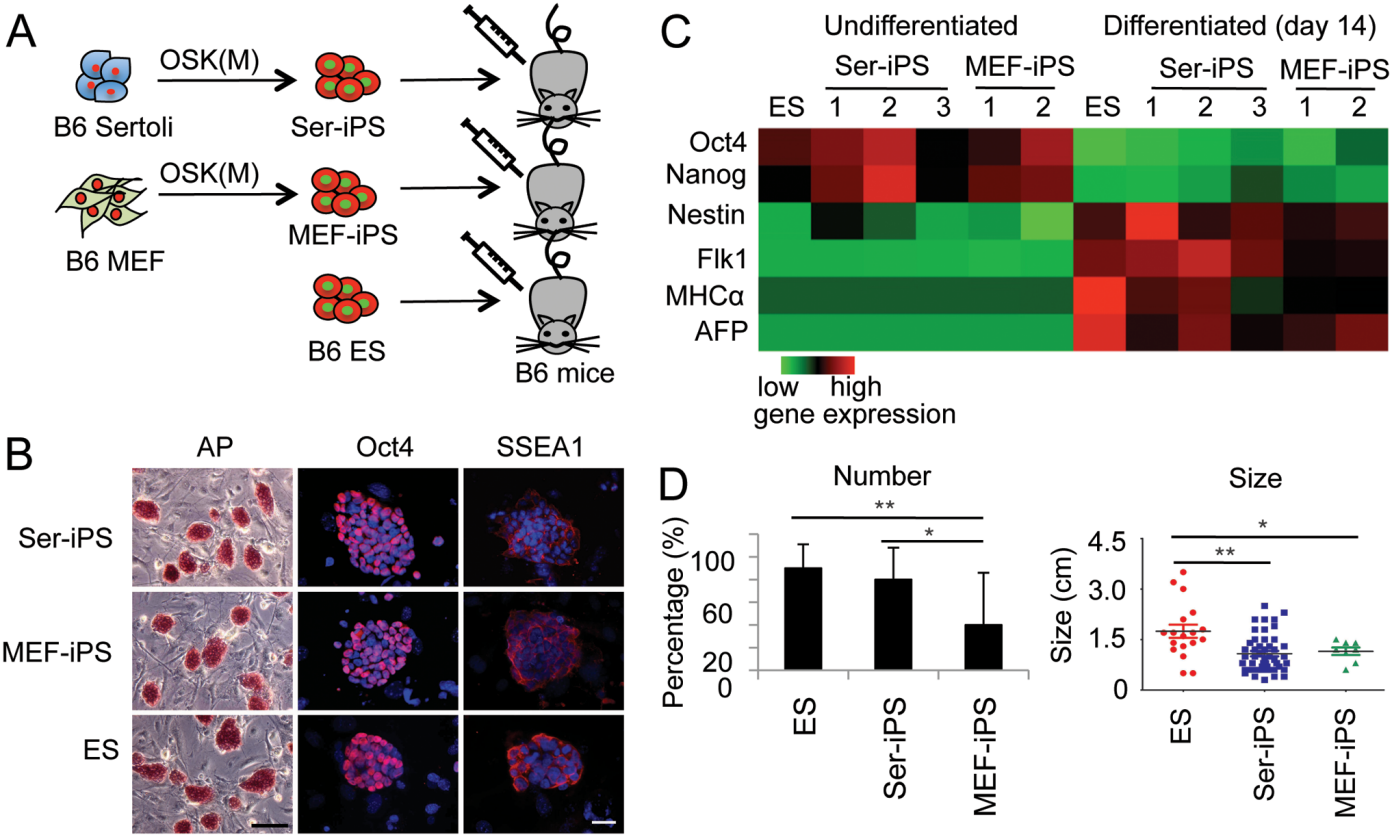
Ser-iPS cells form more teratomas than MEF-iPS cells. (A) Schematic representation of Ser-iPS cell generation from B6 Sertoli cells by Oct4, Sox2 and Klf4 transfection with and without c-Myc (OSKM or OSK) and teratoma assay in B6 mice. (B) AP staining and analysis of pluripotency markers (Oct4 and SSEA1, red). DAPI staining, blue. Scale bars, 200 µm and 35 µm, respectively. AP staining, Ser-iPS cells (OSKM, clone 1), MEF-iPS cells (OSKM, clone 1) and ES cells (JM8). Oct4 and SSEA1 staining, Ser-iPS cells (OSK, clone 3), MEF-iPS cells (OSK, clone 2) and ES cells (JM8). iPS cell data are representative of all Ser-iPS cells and MEF-iPS cells analyzed. (C) Gene expression in undifferentiated and differentiated Ser-iPS cells (EB assays) determined by qRT-PCR is shown in heatmap format. Expression in undifferentiated ES cells (JM8) was arbitrarily set to 1. Ser-iPS 1 refers to 4F iPS cells (OSKM, clone 1) and Ser-iPS 2 and 3 to 3F iPS cells (OSK, clones 2 and 3); MEF-iPS 1 and 2 refer to 4F and 3F iPS cells (OSKM, clone 1 and OSK, clone 2, respectively). (D) Number and size of teratomas of Ser-iPS cells in B6 mice. B6 MEF-iPS cells and B6 ES cells are shown as controls. Average values of Ser-iPS cells (clones 1, 2 and 3) and MEF-iPS cells (clones 1 and 2) are shown. All Ser-iPS cells and MEF-iPS cells are passage 9–15 (early-passage). *P<0.05, **P<0.01. Bars represent mean ± standard deviation.

To generate iPS cells, Sertoli cells were infected with retroviruses expressing three or four reprogramming transcription factors (OSK or OSKM, respectively; Figure 1A) as described [2], [4], [22]. Ser-iPS cells showed typical ES cell morphology, were AP positive and expressed the pluripotency markers Oct4, SSEA1, Sox2, Nanog, Rex1, Rex3 and Dppa4 (Figure 1B, S1C). Three germ layer differentiation potential of Ser-iPS cells was demonstrated by *in* *vitro* EB assay and *in* *vivo* teratoma assay in NOD-SCID mice (Figure 1C, S1D). There was no difference in the frequencies of teratomas formed by Ser-iPS cells, MEF-iPS cells and ES cells. In summary, Ser-iPS cells behaved similar to control MEF-iPS cells and ES cells and thus qualified as bone fide iPS cells.

### Increased teratoma formation by Ser-iPS cells

Immunogenicity of iPS cells and iPS cell-derived cells is detected *in* *vivo* in teratoma assay [7], [28]. Teratomas comprise a broad spectrum of differentiated cells of all three germ layers and thus allow assessing *in* *vivo* immunogenicity of iPS cells and of their differentiated progeny simultaneously. We compared teratoma formation frequency between Ser-iPS cells and MEF-iPS cells by injecting them into syngeneic B6 mice. B6 ES cells were used as a further control (Figure 1A). As expected teratomas of Ser-iPS cells contained cell derivatives of all three germ layers, similar to MEF-iPS cell and ES cell controls (Figure S2A). Importantly, Ser-iPS cells had a much higher incidence of teratoma formation (80%) than MEF-iPS cells (20%) and the incidence of teratoma formation for Ser-iPS cells was similar as for ES cells (90%; Figure 1D). There was no difference in the size of teratomas derived from Ser-iPS cells and MEF-iPS cells, while those of ES cells were larger (Figure 1D). Thus, Ser-iPS cells form more teratomas upon syngeneic transplantation than MEF-iPS cells. These data suggest that iPS cells derived from immune-privileged Sertoli cells elicit less immune responses and thus permit more efficient teratoma formation *in* *vivo*.

### Reduced immunogenicity of syngeneic Ser-iPS cells

**Figure 2:**
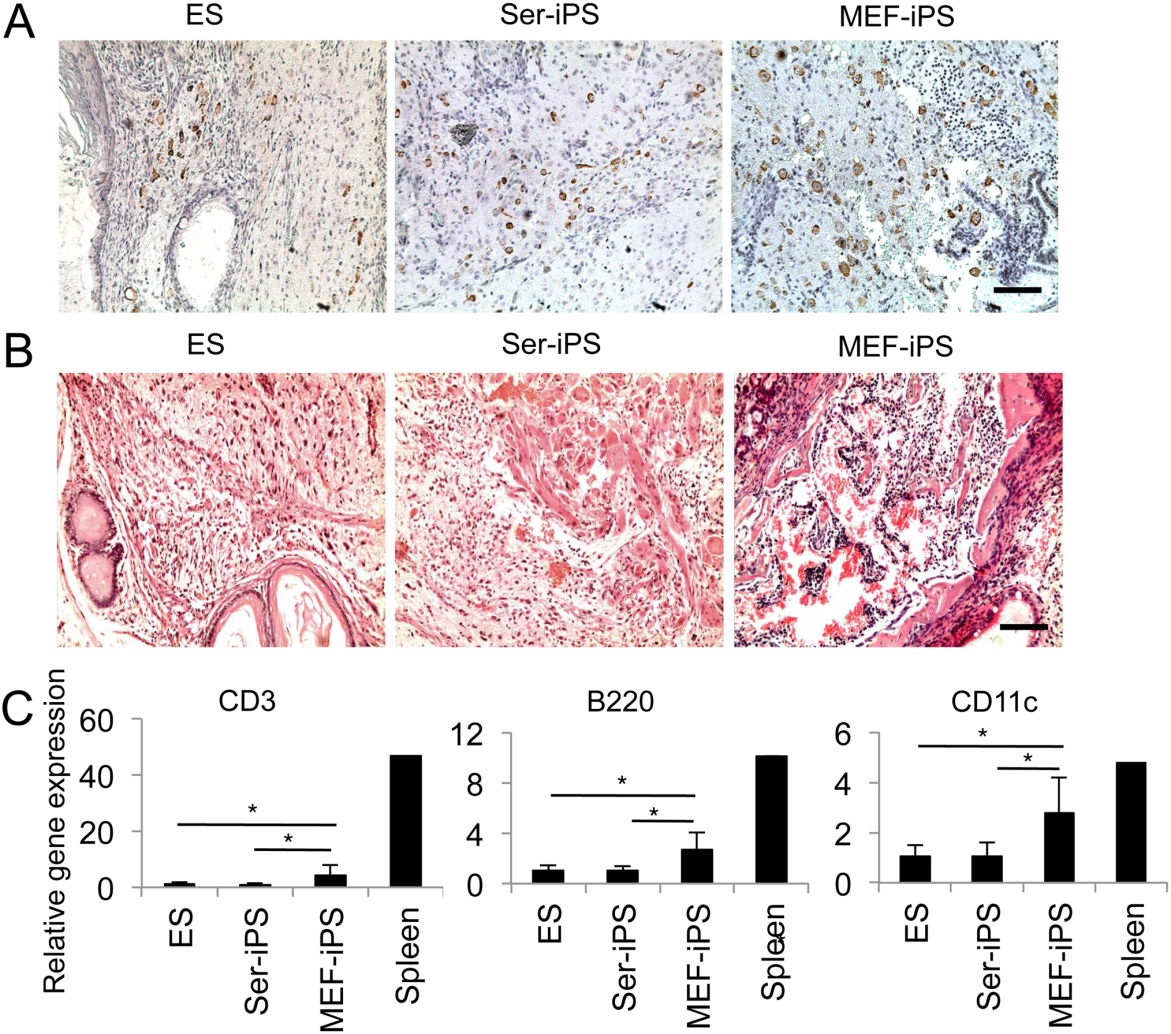
Immunogenicity of syngeneic Ser-iPS cells. (A) CD3 T cell infiltration in Ser-iPS cell teratomas in B6 mice by immunohistochemistry. Teratomas of MEF-iPS cells and ES cells are shown as controls. Ser-iPS cells (OSKM, clone 1), MEF-iPS cells (OSK, clone 2) and ES cells (JM8) of Figure 1C. Images are representative for all Ser-iPS cells and MEF-iPS cells analyzed. CD3 positive T cells, brown. Scale bar, 200 µm. (B) Tissue damage and necrosis are detected in Ser-iPS cell teratomas by HE staining. MEF-iPS cell and ES cell teratomas, controls as in (A). Scale bar, 200 µm. (C) Expression of T cell (CD3), B cell (B220) and DC (CD11c) genes in Ser-iPS cell teratomas by qRT-PCR analysis. MEF-iPS cell and ES cell teratomas, controls as in (A). Average values of Ser-iPS cells (clones 1, 2 and 3) and MEF-iPS cells (clones 1 and 2) are as in Figure 1D. Spleen is shown as a further control. The number of B6 teratomas analyzed: Ser-iPS cells, n = 19; MEF-iPS cells, n = 8; ES cells, n = 10. Relative gene expression was normalized to β-actin. Average mRNA level in ES cell teratomas was arbitrarily set to 1. All Ser-iPS cells and MEF-iPS cells are passage 9–15 (early-passage). *P<0.05. Bars represent mean ± standard deviation.

To further investigate the *in* *vivo* immunogenicity of Ser-iPS cells, we performed immunohistochemical analysis of B6 teratoma sections. As expected, Ser-iPS cell teratomas showed less CD3 T cell infiltration than those of MEF-iPS cells (Figure 2A). Low T cell infiltration of Ser-iPS cell teratomas was similar to syngeneic ES cell teratomas. Additionally, Ser-iPS cell teratomas showed less tissue damage and necrosis than those of MEF-iPS cells (Figure 2B).

We then proceeded to analyze immune cells in teratomas by qRT-PCR. T cell, B cell and dendritic cell (DC) gene expression (CD3, B220 and CD11c, respectively) was lower in Ser-iPS cell teratomas compared to those of MEF-iPS cells (Figure 2C). Expression of CD4 and CD8 T cell markers was also lower in Ser-iPS cell teratomas compared to those of MEF-iPS cells (Figure S2B), however this did not reach statistical significance. There was no significant difference in the expression of macrophage and granulocyte markers (Mac1 and Gr1, respectively).

Expression of Zg16 and Hormad1 genes has been associated with immunogenicity of iPS cells [7], however this finding has remained controversial [9], [10]. Thus, we investigated the expression of Zg16 and Hormad1 in Ser-iPS cell teratomas. Both Ser-iPS cell and MEF-iPS cell teratomas expressed lower level of Zg16 and Hormad1 than ES cell teratomas (Figure S2C).

In summary, Ser-iPS cells have reduced immunogenicity compared to MEF-iPS cells *in* *vivo* and showed lower immune cell infiltration, which is consistent with less tissue damage and necrosis. Additionally, there is apparently no correlation between Zg16 and Hormad1 expression and iPS cell immunogenicity.

### Reduced T cell stimulation potential of differentiating Ser-iPS cells

**Figure 3:**
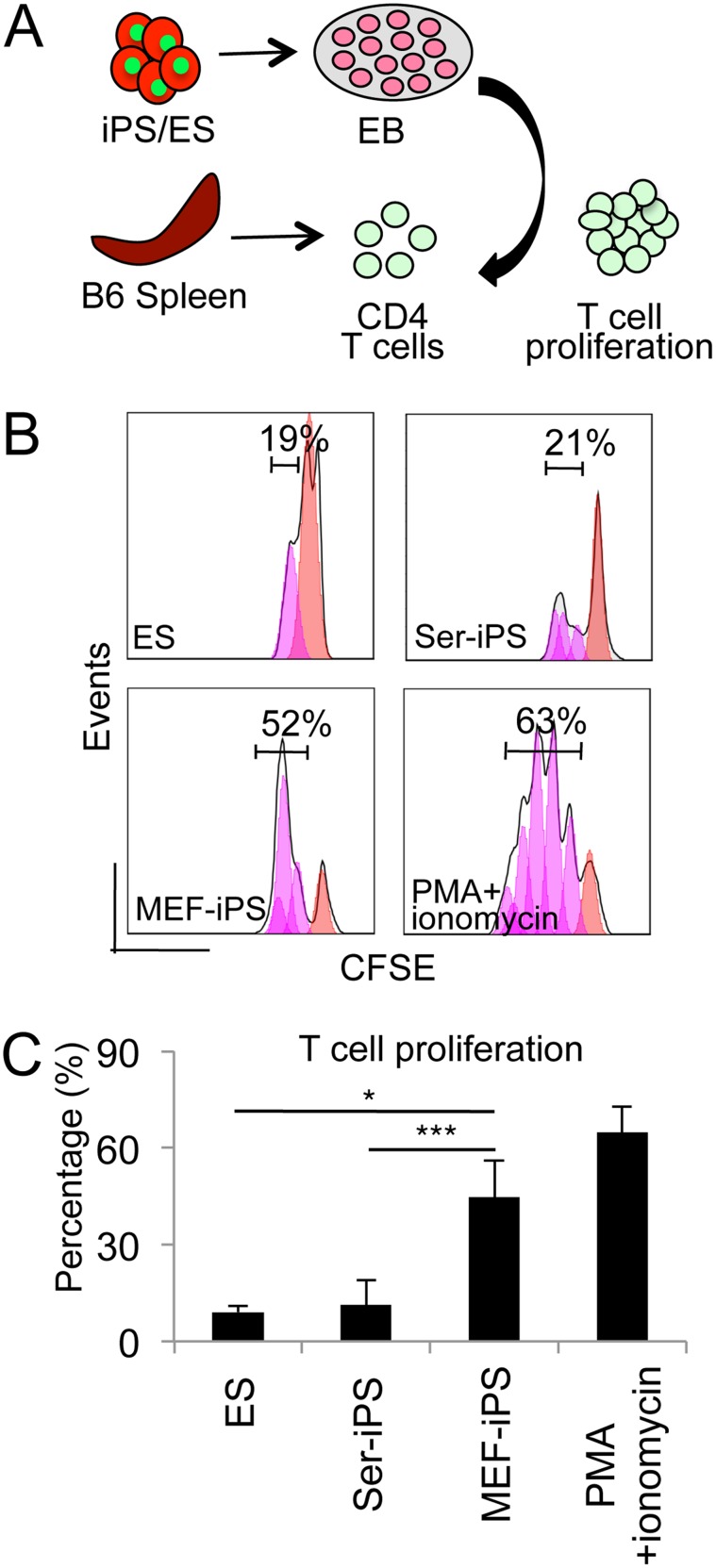
EBs from Ser-iPS cells exhibit reduced CD4 T cell stimulation potential *in* *vitro*. (A) Schematic representation of T cell proliferation assay *in* *vitro*. iPS cells or ES cells were induced to differentiate in EB assays (14 days). EBs were co-cultured with splenic CD4 T cells to assess T cell proliferation. (B) Proliferation of CFSE labeled CD4 T cells co-cultured with Ser-iPS cells (day 12–17) in T cell medium. MEF-iPS cells and ES cells were used as controls. Ser-iPS cells (OSKM, clone 1), MEF-iPS cells (OSKM, clone 1) and ES cells (JM8) of Figure 1C. PMA and ionomycin activated T cells, positive control. T cell proliferation refers to the percentage of dividing T cells after 5 days of co-culture. (C) T cell proliferation data after 5 days of co-culture of (B) (n = 3). Average values of Ser-iPS cells (clones 1, 2 and 3) and MEF-iPS cells (clones 1 and 2) are as in Figure 1D. All Ser-iPS cells and MEF-iPS cells are passage 9–15 (early-passage). *P<0.05; ***P<0.001. Bars represent mean ± standard deviation.

Tissue destruction in teratomas is T cell dependent [7] and Ser-iPS cell teratomas showed low T cell infiltration and tissue damage and necrosis (Figure 2). Thus, we determined the impact of Ser-iPS cells on T cell proliferation *in* *vitro*. EB assay represents the *in* *vitro* equivalent of teratoma formation *in* *vivo*. Therefore, Ser-iPS cells were subjected to EB assays (12 days), dissociated and co-cultured with CD4 T cells (Figure 3A). MEF-iPS cells and ES cells were treated accordingly and used as controls. Ser-iPS cells in EBs stimulated the proliferation of 19% T cells (n = 3), which was significantly lower than that observed for MEF-iPS cells (43%, n = 3), and similar to ES cells (22%, n = 3; Figure 3B, 3C). In co-cultures of T cells with undifferentiated Ser-iPS cells, MEF-iPS cells and ES cells, there was no difference in T cell proliferation (Figure S3A).

Sertoli cells skew T cells towards a Treg profile and this represents one way how Sertoli cells generate an immune-privileged testicular environment [17], [29]. Thus, we examined the frequency of Tregs in co-cultures of CD4 T cells with Ser-iPS cells by measuring the expression of CD4, CD25 and Foxp3 by flow cytometry. MEF-iPS cells and ES cells were used as controls. Sertoli cells were taken as a positive control, since they skew T cells towards a Treg profile [29]. Indeed, Sertoli cells showed an increase in Foxp3 expressing cells (20–26%, Figure S3B). No significant differences were observed for undifferentiated Ser-iPS cells, MEF-iPS cells and ES cells and also for EB-derived differentiated Ser-iPS cells, MEF-iPS cells and ES cells (Figure S3B).

**Figure 4:**
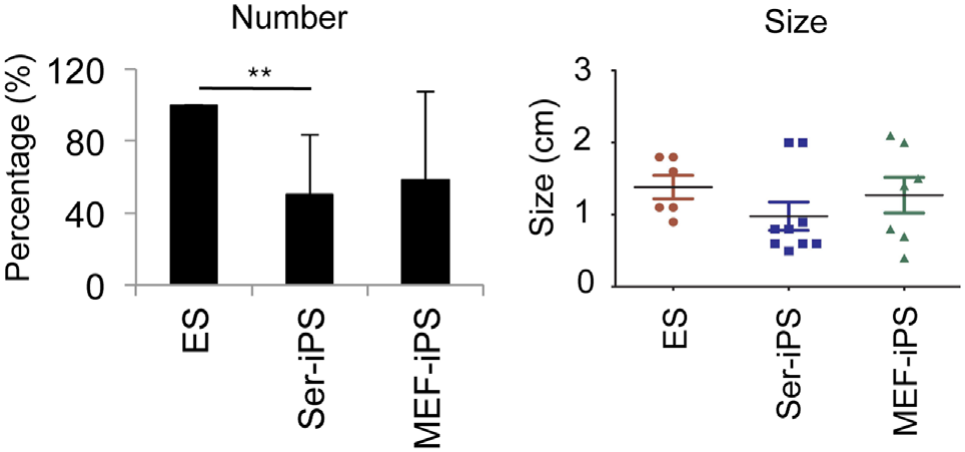
Teratoma formation frequency and teratoma size of late-passage Ser-iPS cells are similar to MEF-iPS cells. Frequency and size of teratomas of late-passage Ser-iPS cells in B6 mice. Late-passage MEF-iPS cells and ES cells are shown as controls. Average values of Ser-iPS cells (clones 1, 2 and 3) and MEF-iPS cells (clones 1 and 2) are as in Figure 1D. Late-passage: p35–38. **P<0.01. There is no statistical difference in teratoma size. Bars represent mean ± standard deviation.

All results obtained so far are based on early-passage iPS cells (p9–15). Therefore, we further investigated whether extended culture periods affect immunogenicity of Ser-iPS cells. We found that the frequency of teratoma formation and teratoma size of late-passage Ser-iPS cells (p35–38) is similar to MEF-iPS cells (Figure 4), indicating that the reduced immunogenicity is lost during extended culture.

Collectively, EBs of Ser-iPS cells exhibit significantly lower T cell stimulation potential *in* *vitro* compared to MEF-iPS cells, which very much relates to the reduced immunogenicity observed in teratoma assays. Yet, Ser-iPS cells apparently do not possess the potential to convert T cells into a Treg phenotype.

## Discussion

In this study we demonstrate that Ser-iPS cells exhibit low immunogenicity both *in* *vivo* and *in* *vitro*. Ser-iPS cells formed more teratomas upon syngeneic transplantation with lower T cell, B cell and DC infiltration and less tissue damage and necrosis than MEF-iPS cells. In addition, EBs formed by Ser-iPS cells possessed lower T cell stimulation potential than MEF-iPS cells. Thus, Ser-iPS cells show reduced immunogenicity and this may be due to some residual somatic memory originating from Sertoli cells used for reprogramming.

Previous studies investigated the immunogenicity of iPS cells and their derivatives following transplantation into syngeneic host. Yet, the immunogenicity of iPS cells and their derived cells has remained highly controversial [7]–[10], [30], [31]. Many factors might influence the immunogenicity of transplanted cells, such as *in* *vitro* culture conditions, cell types used for transplantation and transplantation sites [32]–[34]. Teratoma assay is frequently used to evaluate the immunogenicity of pluripotent stem cells and their derivatives *in* *vivo* [7], [28]. Teratoma contain a variety of differentiated cell types originating from iPS cells and thus provide an useful model for testing the immunogenicity of iPS derivatives [7]. In this assay tumor formation indicates that the recipient’s immune system fails to reject tumor-forming cells [35].

In our study Ser-iPS cells have a stronger capacity to regulate the recipient’s immune response compared to MEF-iPS cells. Ser-iPS cells and/or their differentiated progenies appear to prevent efficient infiltration of host derived immune cells into teratomas resulting in high teratoma formation frequency with less T cell, B cell and DC infiltration. This is consistent with less tissue damage and necrosis in Ser-iPS cell teratomas, since tissue destruction goes along with infiltration of activated T cells [7], [11]. In line with these *in* *vivo* results, EBs of Ser-iPS cells are less potent in stimulating T cell proliferation compared to MEF-iPS cells in *in* *vitro* co-culture experiments. However, in their pluripotent state both Ser-iPS cells and MEF-iPS cells showed T cell responses similar to ES cells. Thus, the immunogenicity of iPS cells was found to be related to the somatic cells used for reprogramming and to the differentiated state.

The immune-privileged function of Sertoli cells includes the generation of Tregs [17], [29]. Therefore, we investigated whether Ser-iPS cell immunogenicity was related to generation of Tregs. Ser-iPS cells showed a Treg profile similar to MEF-iPS cells and ES cells in co-culture experiments. Moreover, Foxp3 expression in teratomas of Ser-iPS cells and MEF-iPS cells was comparable (data not shown). Thus, the reduced immunogenicity of Ser-iPS cells *in* *vitro* and *in* *vivo* appears to be unrelated to the Treg profile.

Several factors have been implicated in the immune-privileged function of Sertoli cells and thus might be responsible for and/or contribute to the reduced immunogenicity of Ser-iPS cells. The anti-inflammatory cytokine transforming growth factor β1 (TGFβ1) and immunosuppressive enzyme indoleamine 2,3-dioxygenase (IDO) produced by Sertoli cells have been reported to protect islet graft in mouse models [16], [36]. However, TGFβ1 and IDO expression was similar in teratomas of Ser-iPS cells and MEF-iPS cells (data not shown). Additionally, expression of interferon γ and IL-4, two cytokines representative for type 1 (destructive) and type 2 (protective) immune responses, respectively, was similar in teratomas of Ser-iPS cells and MEF-iPS cells (data not shown).

Sertoli cell immune function comprises further factors, such as multiple cytokines, chemokines, anti-inflammatory modulators, complement activation inhibitors and adhesion molecules [17]. In light of the complex mechanisms involved in Sertoli cell immune function, it is probably not surprising that we did not find a single molecule or factor, which was important for Ser-iPS cell immunogenicity. Further investigation is warranted to elucidate the mechanisms involved in Sertoli cell immune function and how they contribute to the reduced immunogenicity of Ser-iPS cells.

Two genes, zymogen granule protein 16 (Zg16) and Hormad1, which were expressed in regressing iPS cell teratomas but not in ES cell teratomas, were reported to contribute to iPS cell immunogenicity [7]. However, this observation has remained controversial and was not confirmed in follow up studies [9], [10]. This observation is also in contrast to the result reported here. We did not observe a correlation between Zg16 and Hormad1 expression and iPS cell immunogenicity. Zg16 is highly expressed in pancreas and down-regulated upon injury [37]. Hormad1, also referred to as cancer/testis 46, is involved in chromatin binding and highly expressed in testis and was identified as a tumor antigen [38]. Our results are very much in line with results by Arakia et al. and Guha et al. [9], [10] and Zg16 and Hormad1 expression appears to be not important for the immunogenicity of iPS cells.

The low immunogenicity of Ser-iPS cells was observed in early-passage iPS cells (p9–15). However, late-passage Ser-iPS cells (p35–38) exhibit an immunogenicity similar to the respective MEF-iPS cells. The low immunogenicity in early-passage Ser-iPS cells and its loss in late-passage Ser-iPS cells appear to be consistent with the presence of somatic memory in iPS cells. Somatic memory refers to some remnants of the somatic profile of the cell type used for reprogramming [39]–[45]. Somatic memory impacts on iPS cell properties at early stages [41], [43], [45], and is erased upon continuous *in* *vitro* culture [40], [45], similar to Ser-iPS cell immunogenicity reported here. The exact underlying mechanisms for the loss of Ser-iPS cell reduced immunogenicity during extended passaging have to await further studies.

In summary, we compared the immunogenicity of iPS cells derived from two different somatic cell types, immune-privileged Sertoli cells and MEF. Both Ser-iPS cells and MEF-iPS cells, and the cells derived thereof, showed immunogenicity. Additionally, Ser-iPS cells exhibited reduced immunogenicity compared to MEF-iPS cells, which however got lost upon extended *in* *vitro* culture. These findings indicate that the somatic cell type impacts on the immunogenicity of respective iPS cells. Our results reinforce the concept of using immune-privileged somatic cells for iPS cell generation and derivation of differentiated progeny for transplantation.

## Supporting Information

Figure S1 Ser-iPS cells are pluripotent. (A) Sertoli cells from day 7–10 B6 mice in culture. Phase contrast image at passage 1. Scale bar, 800 µm. (B) RT-PCR analysis of Sertoli cells in (A). Markers for germ cells, spermatogonial stem cells, Leydig cells and Sertoli cells are shown as indicated. Loading control, GAPDH. (C) RT-PCR analysis of pluripotency genes (Oct4, Sox2, Nanog, Rex1, Rex3 and Dppa4) in Ser-iPS clones as in Figure 1C. MEF-iPS cells and ES cells are shown as controls. Loading control, GAPDH. Negative control, MEF. (D) Teratomas of Ser-iPS cells in NOD-SCID mice. Teratomas of MEF-iPS cells and ES cells are shown as controls. Representative images of tissue sections of ectoderm (neural tissue), mesoderm (cartilage) and endoderm (glandular epithelium) from Ser-iPS cells (OSK, clone 3) and MEF-iPS cells (OSKM, clone 1) are shown (HE staining). Images are representative for all Ser-iPS cells and MEF-iPS cells analyzed. All Ser-iPS cells and MEF-iPS cells are passage 9–15 (early-passage). Scale bar, 200 µm.
(TIF)

Figure S2 Ser-iPS cell teratoma formation in B6 mice. (A) Teratomas of Ser-iPS cells in B6 mice. Controls, teratomas of MEF-iPS cells and ES cells. Representative images of tissue sections of ectoderm, mesoderm and endoderm from Ser-iPS cells (OSKM, clone 1), MEF-iPS cells (OSK, clone 2) are shown as in Figure S1D. Images are representative for all Ser-iPS cells and MEF-iPS cells analyzed. Scale bar, 200 µm. (B) Expression of T cell (CD4, CD8), macrophage (Mac), granulocyte (Gr1) genes in teratomas of Ser-iPS cells by qRT-PCR analysis. Teratomas of MEF-iPS cells and ES cells are shown as controls. Spleen is shown as a further control. Relative gene expression is normalized to β-actin. Average mRNA level in ES cell teratomas is arbitrarily set to 1. The number of B6 teratomas analyzed: Ser-iPS cells, n = 19; MEF-iPS cells, n = 8; ES cells, n = 10. Bars represent mean ± standard deviation. (C): Expression of Zg16 and Hormad1 genes in teratomas generated from Ser-iPS cells, MEF-iPS cells and ES cells by qRT-PCR analysis. Relative gene expression was normalized to β-actin as in (B). mRNA levels in MEF were arbitrarily set to 1. Ser-iPS cells and MEF-iPS cells in B and C refer to average values as in Figure 1D. All Ser-iPS cells and MEF-iPS cells are passage 9–15 (early-passage). *P<0.05. Bars represent mean ± standard deviation.
(TIF)

Figure S3 T cell proliferation and Treg profile during co-culture of CD4 T cells with Ser-iPS cells. (A) Proliferation of CD4 T cells co-cultured with Ser-iPS cells (day 0–5) in T cell medium. MEF-iPS cells and ES cells were used as controls. PMA and ionomycin activated T cells, positive control. T cell proliferation refers to the percentage of dividing T cells after 5 days of co-culture (n = 3) as in Figure 3. Bars represent mean ± standard deviation. (B) Treg profile of CD4 T cells after co-culture with Ser-iPS cells (day 0–5) in T cell medium (n = 2, left panel) or after co-culture with EBs of Ser-iPS cells (day 12–17) (n = 2, right panel). T cells were collected after 5 days of co-culture and stained with CD4, CD25 and Foxp3. The gate was set on CD4^+^ cells followed by CD25^+^ cells and Foxp3^+^ cells. T cells without treatment were used as a negative control (T). MEF-iPS cells and ES cells were used as controls as in (A). Sertoli cells are shown as a positive control. Ser-iPS cells and MEF-iPS cells in A and B refer to average values as in Figure 1D. All Ser-iPS cells and MEF-iPS cells are passage 9–15 (early-passage). Bars represent mean ± standard deviation.
(TIF)

Table S1 Primers of qRT-PCR and RT-PCR.
(PDF)

## References

[1] Robinton DA, Daley GQ (2012) The promise of induced pluripotent stem cells in research and therapy. Nature 481: 295–305. 22258608 10.1038/nature10761 PMC3652331

[2] Takahashi K, Yamanaka S (2006) Induction of pluripotent stem cells from mouse embryonic and adult fibroblast cultures by defined factors. Cell 126: 663–676. 16904174 10.1016/j.cell.2006.07.024

[3] Hanna J, Markoulaki S, Schorderet P, Carey BW, Beard C, et al (2008) Direct reprogramming of terminally differentiated mature B lymphocytes to pluripotency. Cell 133: 250–264. 18423197 10.1016/j.cell.2008.03.028 PMC2615249

[4] Kim JB, Sebastiano V, Wu G, Arauzo-Bravo MJ, Sasse P, et al (2009) Oct4-induced pluripotency in adult neural stem cells. Cell 136: 411–419. 19203577 10.1016/j.cell.2009.01.023

[5] Liu H, Ye Z, Kim Y, Sharkis S, Jang YY (2010) Generation of endoderm-derived human induced pluripotent stem cells from primary hepatocytes. Hepatology 51: 1810–1819. 20432258 10.1002/hep.23626 PMC2925460

[6] Nishikawa S, Goldstein RA, Nierras CR (2008) The promise of human induced pluripotent stem cells for research and therapy. Nat Rev Mol Cell Biol 9: 725–729. 18698329 10.1038/nrm2466

[7] Zhao T, Zhang ZN, Rong Z, Xu Y (2011) Immunogenicity of induced pluripotent stem cells. Nature 474: 212–215. 21572395 10.1038/nature10135

[8] de Almeida PE, Meyer EH, Kooreman NG, Diecke S, Dey D, et al (2014) Transplanted terminally differentiated induced pluripotent stem cells are accepted by immune mechanisms similar to self-tolerance. Nat Commun 5: 3903. 24875164 10.1038/ncomms4903 PMC4075468

[9] Guha P, Morgan JW, Mostoslavsky G, Rodrigues NP, Boyd AS (2013) Lack of immune response to differentiated cells derived from syngeneic induced pluripotent stem cells. Cell Stem Cell 12: 407–412. 23352605 10.1016/j.stem.2013.01.006

[10] Araki R, Uda M, Hoki Y, Sunayama M, Nakamura M, et al (2013) Negligible immunogenicity of terminally differentiated cells derived from induced pluripotent or embryonic stem cells. Nature 494: 100–104. 23302801 10.1038/nature11807

[11] Boyd AS, Rodrigues NP, Lui KO, Fu X, Xu Y (2012) Concise review: Immune recognition of induced pluripotent stem cells. Stem Cells 30: 797–803. 22419544 10.1002/stem.1066

[12] Liu P, Chen S, Li X, Qin L, Huang K, et al (2013) Low immunogenicity of neural progenitor cells differentiated from induced pluripotent stem cells derived from less immunogenic somatic cells. PLoS One 8: e69617. 23922758 10.1371/journal.pone.0069617 PMC3724937

[13] Head JR, Neaves WB, Billingham RE (1983) Immune privilege in the testis. I. Basic parameters of allograft survival. Transplantation 36: 423–431. 6353710 10.1097/00007890-198310000-00014

[14] Whitmore WF 3rd, Karsh L, Gittes RF (1985) The role of germinal epithelium and spermatogenesis in the privileged survival of intratesticular grafts. J Urol 134: 782–786. 2863395 10.1016/s0022-5347(17)47438-6

[15] Shamekh R, El-Badri NS, Saporta S, Pascual C, Sanberg PR, et al (2006) Sertoli cells induce systemic donor-specific tolerance in xenogenic transplantation model. Cell Transplant 15: 45–53. 16700329 10.3727/000000006783982205

[16] Suarez-Pinzon W, Korbutt GS, Power R, Hooton J, Rajotte RV, et al (2000) Testicular sertoli cells protect islet beta-cells from autoimmune destruction in NOD mice by a transforming growth factor-beta1-dependent mechanism. Diabetes 49: 1810–1818. 11078447 10.2337/diabetes.49.11.1810

[17] Doyle TJ, Kaur G, Putrevu SM, Dyson EL, Dyson M, et al (2012) Immunoprotective properties of primary Sertoli cells in mice: potential functional pathways that confer immune privilege. Biol Reprod 86: 1–14. 10.1095/biolreprod.110.089425 PMC3313662 21900683

[18] Mital P, Kaur G, Dufour JM (2010) Immunoprotective sertoli cells: making allogeneic and xenogeneic transplantation feasible. Reproduction 139: 495–504. 19995832 10.1530/REP-09-0384

[19] Dym M, Fawcett DW (1970) The blood-testis barrier in the rat and the physiological compartmentation of the seminiferous epithelium. Biol Reprod 3: 308–326. 4108372 10.1093/biolreprod/3.3.308

[20] Mital P, Hinton BT, Dufour JM (2011) The blood-testis and blood-epididymis barriers are more than just their tight junctions. Biol Reprod 84: 851–858. 21209417 10.1095/biolreprod.110.087452 PMC4574632

[21] Yang Y, Han C (2010) GDNF stimulates the proliferation of cultured mouse immature Sertoli cells via its receptor subunit NCAM and ERK1/2 signaling pathway. BMC Cell Biol 11: 78. 20955573 10.1186/1471-2121-11-78 PMC2967512

[22] Nakagawa M, Koyanagi M, Tanabe K, Takahashi K, Ichisaka T, et al (2008) Generation of induced pluripotent stem cells without Myc from mouse and human fibroblasts. Nat Biotechnol 26: 101–106. 18059259 10.1038/nbt1374

[23] Ahmed EA, Barten-van Rijbroek AD, Kal HB, Sadri-Ardekani H, Mizrak SC, et al (2009) Proliferative activity in vitro and DNA repair indicate that adult mouse and human Sertoli cells are not terminally differentiated, quiescent cells. Biol Reprod 80: 1084–1091. 19164176 10.1095/biolreprod.108.071662

[24] Lifschitz-Mercer B, Elliott DJ, Issakov J, Leider-Trejo L, Schreiber L, et al (2002) Localization of a specific germ cell marker, DAZL1, in testicular germ cell neoplasias. Virchows Arch 440: 387–391. 11956819 10.1007/s004280100528

[25] Toyooka Y, Tsunekawa N, Takahashi Y, Matsui Y, Satoh M, et al (2000) Expression and intracellular localization of mouse Vasa-homologue protein during germ cell development. Mech Dev 93: 139–149. 10781947 10.1016/s0925-4773(00)00283-5

[26] Guan K, Nayernia K, Maier LS, Wagner S, Dressel R, et al (2006) Pluripotency of spermatogonial stem cells from adult mouse testis. Nature 440: 1199–1203. 16565704 10.1038/nature04697

[27] Ma X, Dong Y, Matzuk MM, Kumar TR (2004) Targeted disruption of luteinizing hormone beta-subunit leads to hypogonadism, defects in gonadal steroidogenesis, and infertility. Proc Natl Acad Sci USA 101: 17294–17299. 15569941 10.1073/pnas.0404743101 PMC535369

[28] Koch CA, Geraldes P, Platt JL (2008) Immunosuppression by embryonic stem cells. Stem Cells 26: 89–98. 17962705 10.1634/stemcells.2007-0151

[29] Dal Secco V, Riccioli A, Padula F, Ziparo E, Filippini A (2008) Mouse Sertoli cells display phenotypical and functional traits of antigen-presenting cells in response to interferon gamma. Biol Reprod 78: 234–242. 17989360 10.1095/biolreprod.107.063578

[30] Morizane A, Doi D, Kikuchi T, Okita K, Hotta A, et al (2013) Direct comparison of autologous and allogeneic transplantation of iPSC-derived neural cells in the brain of a nonhuman primate. Stem Cell Reports 1: 283–292. 24319664 10.1016/j.stemcr.2013.08.007 PMC3849265

[31] Okita K, Nagata N, Yamanaka S (2011) Immunogenicity of induced pluripotent stem cells. Circ Res 109: 720–721. 21921270 10.1161/RES.0b013e318232e187

[32] Cao J, Li X, Lu X, Zhang C, Yu H, et al (2014) Cells derived from iPSC can be immunogenic - yes or no? Protein Cell 5: 1–3. 24474200 10.1007/s13238-013-0003-2 PMC3938852

[33] Scheiner ZS, Talib S, Feigal EG (2014) The potential for immunogenicity of autologous induced pluripotent stem cell-derived therapies. J Biol Chem 289: 4571–4577. 24362036 10.1074/jbc.R113.509588 PMC3931018

[34] Fu X (2014) The immunogenicity of cells derived from induced pluripotent stem cells. Cell Mol Immunol 11: 14–16. 24336164 10.1038/cmi.2013.60 PMC4002141

[35] Dressel R (2011) Effects of histocompatibility and host immune responses on the tumorigenicity of pluripotent stem cells. Semin Immunopathol 33: 573–591. 21461989 10.1007/s00281-011-0266-8 PMC3204002

[36] Fallarino F, Luca G, Calvitti M, Mancuso F, Nastruzzi C, et al (2009) Therapy of experimental type 1 diabetes by isolated Sertoli cell xenografts alone. J Exp Med 206: 2511–2526. 19822646 10.1084/jem.20090134 PMC2768846

[37] Neuschwander-Tetri BA, Fimmel CJ, Kladney RD, Wells LD, Talkad V (2004) Differential expression of the trypsin inhibitor SPINK3 mRNA and the mouse ortholog of secretory granule protein ZG-16p mRNA in the mouse pancreas after repetitive injury. Pancreas 28: e104–111. 15097871 10.1097/00006676-200405000-00022

[38] Chen YT, Venditti CA, Theiler G, Stevenson BJ, Iseli C, et al (2005) Identification of CT46/HORMAD1, an immunogenic cancer/testis antigen encoding a putative meiosis-related protein. Cancer Immun 5: 9. 15999985

[39] Bar-Nur O, Russ HA, Efrat S, Benvenisty N (2011) Epigenetic memory and preferential lineage-specific differentiation in induced pluripotent stem cells derived from human pancreatic islet beta cells. Cell Stem Cell 9: 17–23. 21726830 10.1016/j.stem.2011.06.007

[40] Chin MH, Pellegrini M, Plath K, Lowry WE (2010) Molecular analyses of human induced pluripotent stem cells and embryonic stem cells. Cell Stem Cell 7: 263–269. 20682452 10.1016/j.stem.2010.06.019 PMC3276111

[41] Kim K, Doi A, Wen B, Ng K, Zhao R, et al (2010) Epigenetic memory in induced pluripotent stem cells. Nature 467: 285–290. 20644535 10.1038/nature09342 PMC3150836

[42] Kim K, Zhao R, Doi A, Ng K, Unternaehrer J, et al (2011) Donor cell type can influence the epigenome and differentiation potential of human induced pluripotent stem cells. Nat Biotechnol 29: 1117–1119. 22119740 10.1038/nbt.2052 PMC3357310

[43] Ohi Y, Qin H, Hong C, Blouin L, Polo JM, et al (2011) Incomplete DNA methylation underlies a transcriptional memory of somatic cells in human iPS cells. Nat Cell Biol 13: 541–549. 21499256 10.1038/ncb2239 PMC3987913

[44] Pfaff N, Lachmann N, Kohlscheen S, Sgodda M, Arauzo-Bravo MJ, et al (2012) Efficient hematopoietic redifferentiation of induced pluripotent stem cells derived from primitive murine bone marrow cells. Stem Cells Dev 21: 689–701. 21732815 10.1089/scd.2011.0010

[45] Polo JM, Liu S, Figueroa ME, Kulalert W, Eminli S, et al (2010) Cell type of origin influences the molecular and functional properties of mouse induced pluripotent stem cells. Nat Biotechnol 28: 848–855. 20644536 10.1038/nbt.1667 PMC3148605

