# Supplementary figures and images for: Reduced Immunogenicity of Induced Pluripotent Stem Cells Derived from Sertoli Cells
Source: PLoS One. 2014 Aug 28;9(8):e106110. doi: 10.1371/journal.pone.0106110 (PMC4148392; doi:10.1371/journal.pone.0106110)

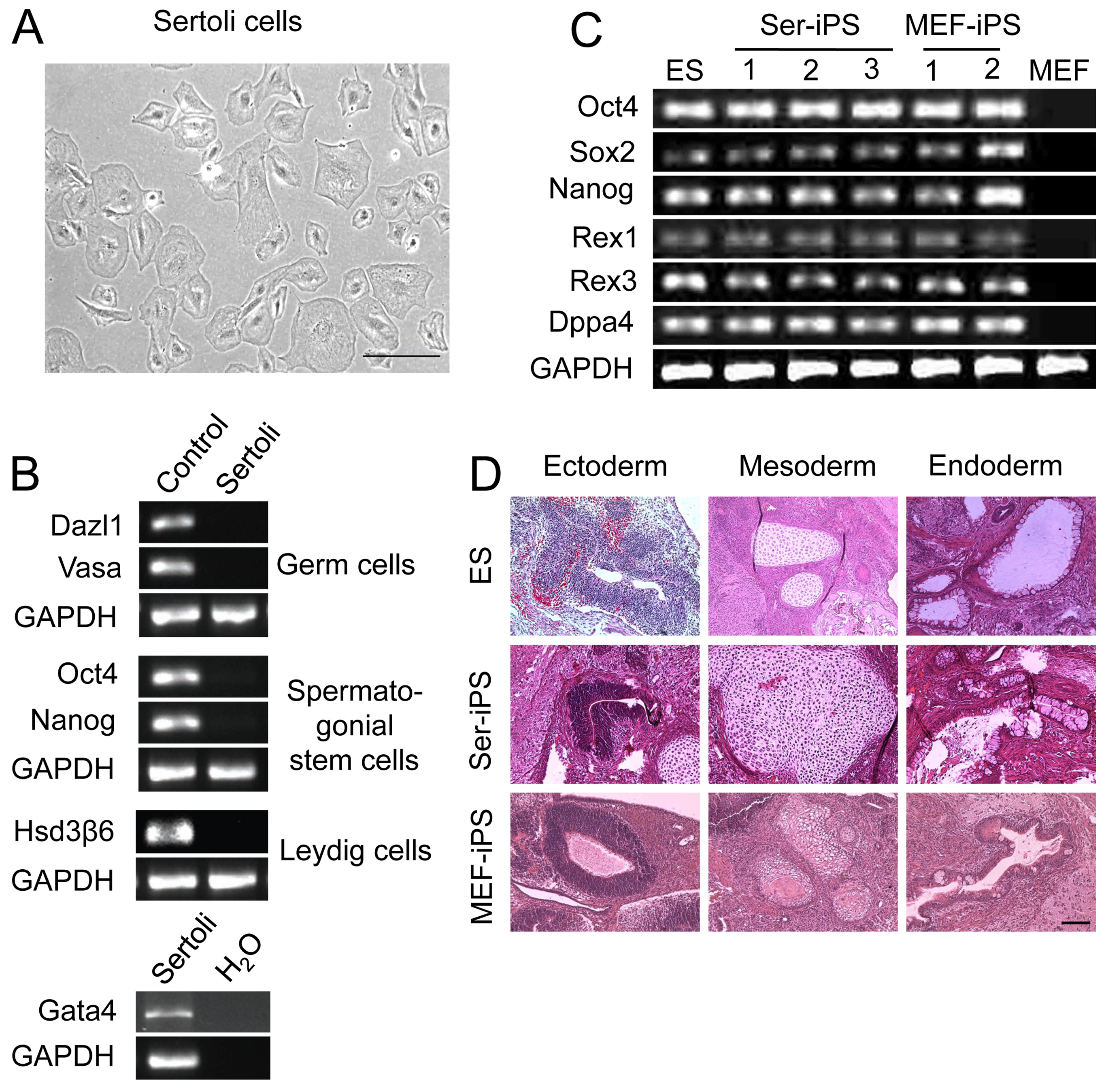

Supplement: Figure S1 — Ser-iPS cells are pluripotent. (A) Sertoli cells from day 7–10 B6 mice in culture. Phase contrast image at passage 1. Scale bar, 800 µm. (B) RT-PCR analysis of Sertoli cells in (A). Markers for germ cells, spermatogonial stem cells, Leydig cells and Sertoli cells are shown as indicated. Loading control, GAPDH. (C) RT-PCR analysis of pluripotency genes (Oct4, Sox2, Nanog, Rex1, Rex3 and Dppa4) in Ser-iPS clones as in Figure 1C. MEF-iPS cells and ES cells are shown as controls. Loading control, GAPDH. Negative control, MEF. (D) Teratomas of Ser-iPS cells in NOD-SCID mice. Teratomas of MEF-iPS cells and ES cells are shown as controls. Representative images of tissue sections of ectoderm (neural tissue), mesoderm (cartilage) and endoderm (glandular epithelium) from Ser-iPS cells (OSK, clone 3) and MEF-iPS cells (OSKM, clone 1) are shown (HE staining). Images are representative for all Ser-iPS cells and MEF-iPS cells analyzed. All Ser-iPS cells and MEF-iPS cells are passage 9–15 (early-passage). Scale bar, 200 µm. (TIF) [file pone.0106110.s001.tif]

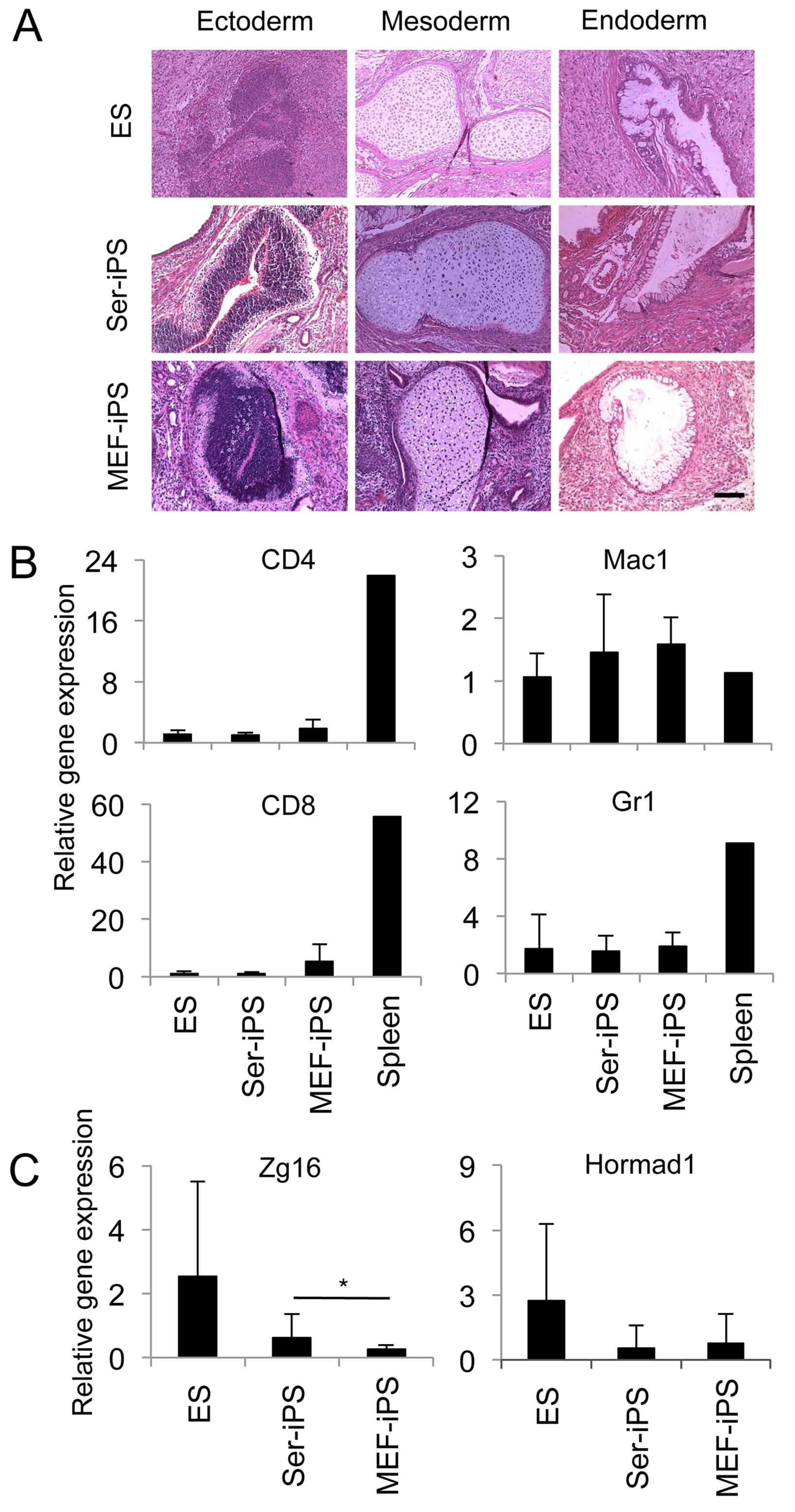

Supplement: Figure S2 — Ser-iPS cell teratoma formation in B6 mice. (A) Teratomas of Ser-iPS cells in B6 mice. Controls, teratomas of MEF-iPS cells and ES cells. Representative images of tissue sections of ectoderm, mesoderm and endoderm from Ser-iPS cells (OSKM, clone 1), MEF-iPS cells (OSK, clone 2) are shown as in Figure S1D. Images are representative for all Ser-iPS cells and MEF-iPS cells analyzed. Scale bar, 200 µm. (B) Expression of T cell (CD4, CD8), macrophage (Mac), granulocyte (Gr1) genes in teratomas of Ser-iPS cells by qRT-PCR analysis. Teratomas of MEF-iPS cells and ES cells are shown as controls. Spleen is shown as a further control. Relative gene expression is normalized to β-actin. Average mRNA level in ES cell teratomas is arbitrarily set to 1. The number of B6 teratomas analyzed: Ser-iPS cells, n = 19; MEF-iPS cells, n = 8; ES cells, n = 10. Bars represent mean ± standard deviation. (C): Expression of Zg16 and Hormad1 genes in teratomas generated from Ser-iPS cells, MEF-iPS cells and ES cells by qRT-PCR analysis. Relative gene expression was normalized to β-actin as in (B). mRNA levels in MEF were arbitrarily set to 1. Ser-iPS cells and MEF-iPS cells in B and C refer to average values as in Figure 1D. All Ser-iPS cells and MEF-iPS cells are passage 9–15 (early-passage). *P<0.05. Bars represent mean ± standard deviation. (TIF) [file pone.0106110.s002.tif]

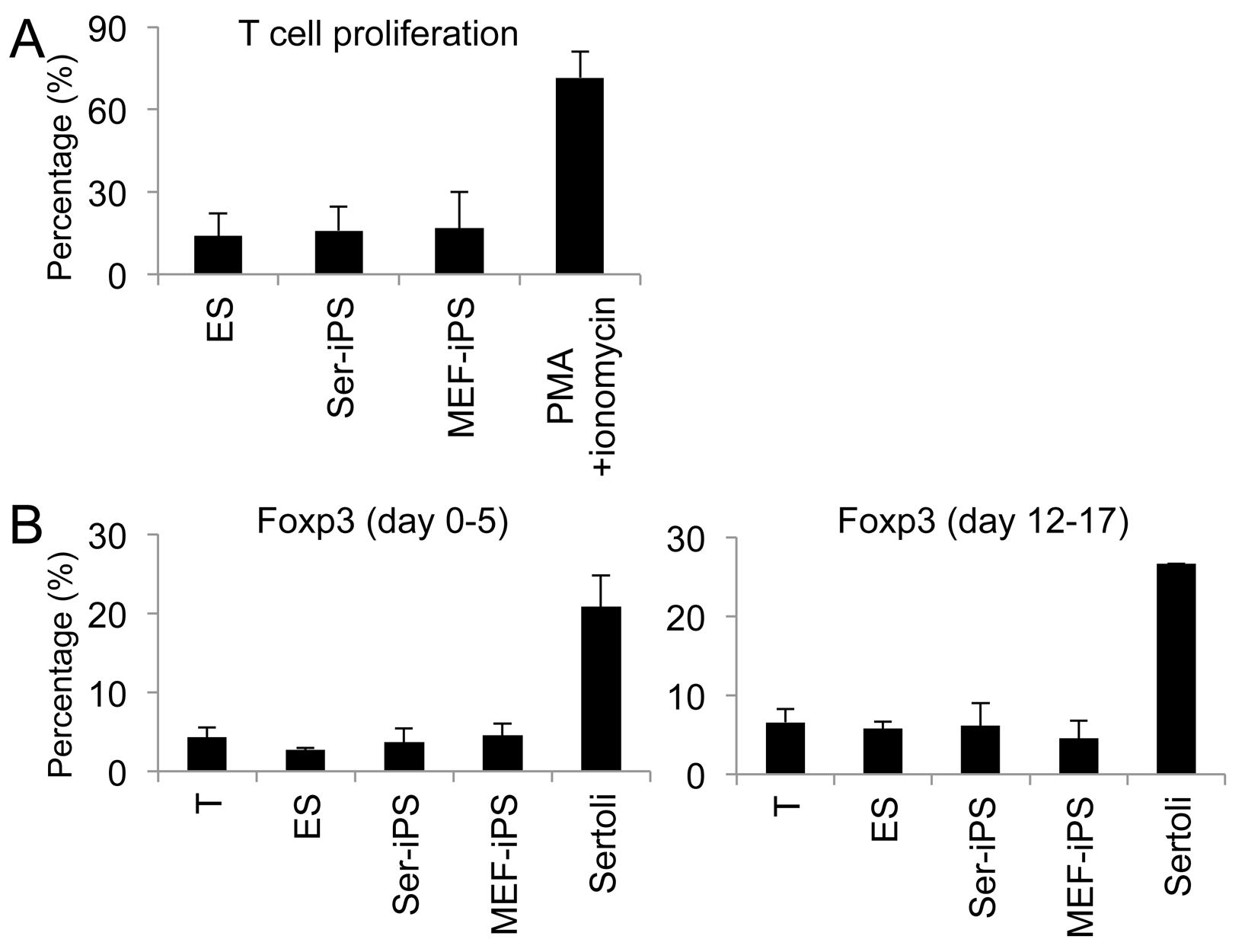

Supplement: Figure S3 — T cell proliferation and Treg profile during co-culture of CD4 T cells with Ser-iPS cells. (A) Proliferation of CD4 T cells co-cultured with Ser-iPS cells (day 0–5) in T cell medium. MEF-iPS cells and ES cells were used as controls. PMA and ionomycin activated T cells, positive control. T cell proliferation refers to the percentage of dividing T cells after 5 days of co-culture (n = 3) as in Figure 3. Bars represent mean ± standard deviation. (B) Treg profile of CD4 T cells after co-culture with Ser-iPS cells (day 0–5) in T cell medium (n = 2, left panel) or after co-culture with EBs of Ser-iPS cells (day 12–17) (n = 2, right panel). T cells were collected after 5 days of co-culture and stained with CD4, CD25 and Foxp3. The gate was set on CD4+ cells followed by CD25+ cells and Foxp3+ cells. T cells without treatment were used as a negative control (T). MEF-iPS cells and ES cells were used as controls as in (A). Sertoli cells are shown as a positive control. Ser-iPS cells and MEF-iPS cells in A and B refer to average values as in Figure 1D. All Ser-iPS cells and MEF-iPS cells are passage 9–15 (early-passage). Bars represent mean ± standard deviation. (TIF) [file pone.0106110.s003.tif]
