# Supplementary material for: Reduced Immunogenicity of Induced Pluripotent Stem Cells Derived from Sertoli Cells
Source: PLoS One. 2014 Aug 28;9(8):e106110. doi: 10.1371/journal.pone.0106110 (PMC4148392; doi:10.1371/journal.pone.0106110)
Supplement: Table S1 — Primers of qRT-PCR and RT-PCR. (PDF) [file pone.0106110.s004.pdf]

Supporting Information 2

Table S1: Primers of qRT-PCR and RT-PCR

Primer sequences for qRT-PCR

Gene	Primer Sequence	Reference	Gene	Primer Sequence	Reference
Oct4	F-TCTTTCCACCAGGCCCCCGGCTC	[1]	CD4	F- CAAGCGCCTAAGAGAGATGG	
	R-TGCGGGCGGACATGGGGAGATCC			R- CACCTGTGCAAGAAGCAGAG	
Nanog	F-CAGGTGTTTGAGGGTAGCTC	[1]	CD8b	F- GATGGTCTTTGGGACAGGG	[2]
	R-CGGTTCATCATGGTACAGTC			R- ATGCCAGCAGAAGCAGGAT	
Nestin	F-AGAGTCAGATCGCTCAGATCC		CD11c	F- CTGGATAGCCTTTCTTCTGCT	[3]
	R-GCAGAGTCCTGTATGTAGCCAC			R- GCACACTGTGTCCGAACTC	
Flk1	F-CTTCTGGCTCCTTCTTGTCATTG		B220	F- AAGAGTTGTGAGGCTGGCAC	
	R-TCATTGGCCCGCTTAACG			R- GCTCAAACTTCTGGCCTTTG	
MHC α	F-CATGGCATCACTCTTCTCTACCTATGC		Mac1	F- GTTTGTTGAAGGCATTTCCC	
	R-TACCACTGACACCGGTATCA			R- ATTCGGTGATCCCTTGGATT	
AFP	F-TCAGCGAGGAGAAATGGTCC		Gr1	F- TGCCCCTTCTCTGATGGATT	[4]
	R-ATGAAAATGTCGGCCATTCC			R- TGCTCTTGACTTTGCTTCTGTGA	
β-actin	F-CACCAGTTCGCCATGGATG		Zg16	F- CATCACCGCCTTCCGTAT	[5]
	R-GCCGTTGTCGACGACCAG			R- CGTTGAAACTTGTGCCTGA	
CD3	F-TCGTCACTGTCTAGAGGGCA		Hormad1	F- CCAGATTACCAACCACCAG	[5]
	R-CCTCCTAGCTGTTGGCACTT			R- TGAAAAGGTGTTGGGACT	

Primer sequences for RT-PCR

Gene	Primer Sequence	Reference	Gene	Primer Sequence	Reference
Dazl1	F- GCACTCAGTCTTCATCAGCAAC	[6]	Rex1	F- CAGCAGCTCCTGCACACAGA	
	R- CTATCTTCTGCACATCCACGTC			R- GATCCGCAAACACCTGCTTT	
Vasa	F- CTTGCAGAGATGTTCAGCAGAC	[6]	Rex3	F- GAGCGGAGCAGGTCTGAGAA	
	R- CTCCAAGAGCTTGCTCTCTCTC			R- GACTCCATTACTCCTGGGCCT	
Hsd3β6	F- TGGACAAGTTCTTCAGACCAGA		Dppa4	F- CATTGGGGGTAGGAACACGGAAGG	
	R- TCTCCTTCCAACACTGTCACC			R- TGCCCCAAGTGTGTTCATAA	
Gata4	F- CCCTACCCAGCCTACATGG	[7]	GAPDH	F- GGGGTGAGGCCGGTGCTGAGTAT	
	R- GAGCTGGCCTGCGATGTCTGAGTG			R- CATTGGGGGTAGGAACACGGAAGG	
Sox2	F- TAGAGCTAGACTCCGGGCGATGA	[1]			
	R- TTGCCTTAAACAAGACCACGAAA				

All primer sequences are 5'➔3', F: forward primers, R: reverse primers.

References

1. Takahashi K, Yamanaka S (2006) Induction of pluripotent stem cells from mouse embryonic and adult fibroblast cultures by defined factors. Cell 126: 663-676.
2. Rui J, Liu H, Zhu X, Cui Y, Liu X (2012) Epigenetic silencing of CD8 genes by ThPOK-mediated deacetylation during CD4 T cell differentiation. J Immunol 189: 1380-1390.
3. Shaul ME, Bennett G, Strissel KJ, Greenberg AS, Obin MS (2010) Dynamic, M2-like remodeling phenotypes of CD11c+ adipose tissue macrophages during high-fat diet-induced obesity in mice. Diabetes 59: 1171-1181.
4. Doloff JC, Waxman DJ (2012) VEGF receptor inhibitors block the ability of metronomically dosed cyclophosphamide to activate innate immunity-induced tumor regression. Cancer Res 72: 1103-1115.
5. Zhao T, Zhang ZN, Rong Z, Xu Y (2011) Immunogenicity of induced pluripotent stem cells. Nature 474: 212-215.
6. Ko K, Tapia N, Wu G, Kim JB, Bravo MJ, et al. (2009) Induction of pluripotency in adult unipotent germline stem cells. Cell Stem Cell 5: 87-96.
7. Kyronlahti A, Euler R, Bielinska M, Schoeller EL, Moley KH, et al. (2011) GATA4 regulates Sertoli cell function and fertility in adult male mice. Mol Cell Endocrinol 333: 85-95.
